# GC-MS and UHPLC-QTOFMS-assisted identification of the differential metabolites and metabolic pathways in key tissues of *Pogostemon cablin*

**DOI:** 10.3389/fpls.2023.1098280

**Published:** 2023-02-27

**Authors:** Xiaobing Wang, Liting Zhong, Xuan Zou, Lizhen Gong, Jiexuan Zhuang, Danhua Zhang, Hai Zheng, Xiaomin Wang, Daidi Wu, Ruoting Zhan, Likai Chen

**Affiliations:** ^1^ Research Center of Chinese Herbal Resource Science and Engineering, Guangzhou University of Chinese Medicine, Key Laboratory of Chinese Medicinal Resource from Lingnan (Guangzhou University of Chinese Medicine), Ministry of Education, Joint Laboratory of National Engineering Research Center for the Pharmaceutics of Traditional Chinese Medicines, Guangzhou, China; ^2^ School of Pharmaceutical Sciences, Guangdong Food and Drug Vocational College, Guangzhou, China; ^3^ Maoming Branch, Guangdong Laboratory for Lingnan Modern Agriculture, Maoming, Guangdong, China

**Keywords:** metabolism pathway, metabolite biomarker, metabolomics, phytochemical, *Pogostemon cablin*

## Abstract

*Pogostemon cablin* is an important aromatic medicinal herb widely used in the pharmaceutical and perfume industries. However, our understanding of the phytochemical compounds and metabolites within *P. cablin* remains limited. To our knowledge, no integrated studies have hitherto been conducted on the metabolites of the aerial parts of *P. cablin*. In this study, twenty-three volatile compounds from the aerial parts of *P. cablin* were identified by GC-MS, predominantly sesquiterpenes. Quantitative analysis showed the highest level of patchouli alcohol in leaves (24.89 mg/g), which was 9.12 and 6.69-fold higher than in stems and flowers. UHPLC-QTOFMS was used to analyze the non-volatile compounds of leaf, stem and flower tissues. The differences in metabolites between flower and leaf tissues were the largest. Based on 112, 77 and 83 differential metabolites between flower-leaf, flower-stem and leaf-stem, three tissue-specific biomarkers of metabolites were identified, and the differential metabolites were enriched in several KEGG pathways. Furthermore, labeling differential metabolites in the primary and secondary metabolic pathways showed that flowers accumulated more lipids and amino acids, including proline, lysine and tryptophan; the leaves accumulated higher levels of terpenoids, vitamins and flavonoids, and stems contained higher levels of carbohydrate compounds. Based on the role of acetyl coenzyme A, the distribution and possible exchange mechanism of metabolites in leaves, stems and flowers of *P. cablin* were mapped for the first time, laying the groundwork for future research on the metabolites in *P. cablin* and their regulatory role.

## Introduction

*Pogostemon cablin* (*P. cablin*) is a member of the Lamiaceae family, native to tropical areas such as Malaysia, and widely cultivated in Guangdong, Guangxi and Hainan provinces in China (Hu et al., 2006). The aerial parts of *P. cablin* consists of leaves, stems and flowers. The leaves and stems of *P. cablin* have a strong aromatic taste after maceration, substantiating that it is rich in volatile components, such as sesquiterpenes. In addition, leaves and stems are rich in non-volatile substances, such as flavonoids and alkaloids. However, the metabolites in *P. cablin* flowers have been rarely reported since they rarely bloom. *P. cablin* is not only used as the fixative of various perfumes but also as the raw material of various cosmetics and oral hygiene products (Sandes et al., 2016). Interestingly, it is also used in traditional Chinese medicine to treat colds, nausea, diarrhea, headache and fever (Liu et al., 2017), suggesting that the market demand is huge. One study found that there are many active components in *P. cablin* (Hu et al., 2006), and specific compounds have been gradually revealed in recent years, including terpenoids (Chen et al., 2019), flavonoids (Miyazawa et al., 2000), phytosterols, organic acids (Xie et al., 2022), phenols, alkaloids and glycosides (Swamy and Sinniah, 2015). The accumulation and exchange of these metabolites are reportedly closely related to the biological function of *P. cablin*; however, the underlying process remains unknown.

Patchouli alcohol represents a major bioactive and aromatic compound in *P. cablin*, widely used as an indicator of sample quality. Besides, patchouli alcohol is a natural sesquiterpenoid. In plants, terpenoids are usually produced by the mevalonate pathway (MVA) and methylerythritol phosphate (MEP) pathway. The cytoplasmic MVA pathway is the main pathway for synthesizing sesquiterpenes, and its original substrate is acetyl-CoA. It has been established that AACT primarily catalyzes acetyl-CoA to produce acetoacetyl-CoA, which is critical for the biosynthesis of the steroid backbone (Wang et al., 2017). Isopentenyl diphosphate is an intermediate substance linking the MVA and MEP pathways (Henry et al., 2018). The MVA pathway eventually produces sesquiterpenes and triterpenes, while the MEP pathway produces diterpenes and other steroids (Ahmed et al., 2016; Schwarz et al., 2018). However, the tissue-specific differences in the metabolic pathways responsible for synthesizing the active ingredients in *P. cablin* remain unknown.

The past decade has witnessed unprecedented medical advances with the advent of metabolomics which can be harnessed to study the accumulation of medicinally active ingredients in different conditions. Metabolomic analyses rely on research methods, such as gas chromatography-mass spectrometry (GC-MS), liquid chromatography-mass spectrometry (LC-MS), and ultra-high-performance liquid chromatography coupled with quadrupole time-of-flight mass spectrometry (UHPLC-QTOFMS), and each method has unique advantages and disadvantages (Patterson et al., 2010). In a comprehensive study of lettuce metabolites in different cultivation environments using GC-MS and LC-MS, the researchers found that the level of amino acids such as lysine, phenylalanine, tryptophan and valine was significantly increased in hydroponically grown leaf lettuce, while soil-cultivation derived leaf lettuce samples contained significantly higher levels of fatty-acid derived alcohols and lettuce specific sesquiterpene lactones. In addition, the difference in metabolite content endowed lettuce with different nutritional components and tastes (Tamura et al., 2018). Moreover, other vegetables have been subjected to similar analyses. For example, the quality of three different cultivars of cynara scolymus (artichoke) was analyzed; UHPLC and qTOF-MS results showed the content of caffeic acid derivatives, flavonoids and fatty acid was different among cultivars (Farag et al., 2013). Ultrahigh-pressure liquid chromatography-high-resolution mass spectrometry (UHPLC-HRMS) has been used to evaluate the freshness of egg products, and 31 compounds have been identified as useful markers of egg freshness (Cavanna et al., 2018). Metabolomics methods have also been used to study the differences in metabolites produced under stress conditions in different tissues. Interestingly, an integrated GC-MS and LC-MS analysis revealed that disease-resistant tomatoes contained significantly higher concentrations of acyl sugars (Firdaus et al., 2013). Moreover, stress- related substances have been documented on the waxy cuticles of wheat leaves and stems. GC-MS has been used to analyze the metabolite composition of wheat epidermal wax and found that leaves contained more primary alcohol than stems (Lavergne et al., 2018). In addition, an integrated GC-MS and LC-MS metabonomics study of pumpkins showed a significant difference in the chemical composition of male and female nectaries (Chatt et al., 2018). A recent multivariate statistical analysis of Corni Fructus based on UHPLC-QTOFMS identified 17 different compounds between raw and processed products, revealing the relationship between compounds and the color of Corni Fructus and the crucial compounds for color (Qian et al., 2022).

Herein, GC-MS and UHPLC-QTOFMS were used to study the volatile and non-volatile substances in the leaves, stems and flowers of *P. cablin* and their metabolic pathways. We carried out a tissue-specific analysis of the primary and secondary metabolites, and the metabolites in the flowers of this species were analyzed for the first time. Next, metabolomics and pathway analyses were performed to assess the phytochemical panorama and metabolism pathway connectivity. We reported the relative metabolite contents of different tissues and characterized the metabolic networks present in *P. cablin*. Based on the above contents, this study mapped the distribution and possible exchange mechanism of metabolites in key tissues of *P. cablin*, which provides the foothold for further research on metabolites and the regulation of this important aromatic medicinal plant.

## Materials and methods

### Plant materials

*P. cablin* used in this study was collected at the flowering stage from Shizhen Mountain at Guangzhou University of Chinese Medicine (23.03°N, 113.23°E) in Guangdong province, China. The plants were cultured in a growth chamber in our laboratory. For GC-MS analysis, *P. cablin* leaves, stems and flowers were collected and dried in an oven at 40 °C. The same samples were collected again and immediately placed in liquid nitrogenfor UHPLC-QTOFMS analysis. All samples were kept in our laboratory.

### Analysis of volatile compounds in *P. cablin* tissues using GC-MS

0.25 g sample powder of leaves, stems and flowers was accurately weighed. Extracted twice with 50 mL ethyl acetate for 20 min each time using an ultrasonic cleaner, and then concentrate the filtrate by rotating evaporation. The concentrate was dissolved in hexane and diluted in a 5 mL volumetric flask. 0.22 μM organic membranes were used to filter the solution, and the filtrate was transferred to a new sample injection bottle for GC-MS analysis using an Agilent 7890B gas chromatograph with 5977A inert mass selective detector (Agilent, United States). The injection volume was 1 μL, and helium was the carrier gas. The Agilent HP-5MS column (30 m × 0.25 mm × 0.25 μm film thickness) was used for separation. The GC oven temperature was programmed at an initial temperature of 50 °C for 2 min with an increase of 20 °C/min to 130 °C and increased to 150 °C at a rate of 2 °C/min for 5 min. The temperature was then increased to 230°C at 20 °C/min. Quadrupole and ion source temperatures are set to 150 °C and 230 °C. The NIST14/Wiley275 mass spectral library was used for metabolite identification. The external standard method was used to quantify the content of patchouli alcohol in leaves, stems and flowers. Three biological replicates and two technical replicates were performed for each tissue in this study.

### Extraction of non-volatile compounds in *P. cablin* tissues

50 mg samples were extracted from 1 mL of extraction solution containing an internal standard (*V* methanol: *V* acetonitrile: *V* water = 2:1:1, which was kept at -20 °C before extraction). The sample was homogenized in a ball mill for 4 minutes at 45 Hz and then ultrasound treated for 5 minutes (incubated in ice water). After homogenization for 3 times, the sample was incubated at -20 °C for 1 h to precipitate proteins. Then, the sample was centrifuged at 12000 rpm for 15 min at 4 °C and the supernatant (500 μL) was transferred into EP tubes. After drying the extracts in a vacuum concentrator without heating, the sample was reconstituted in 200 μL extraction liquid (*V* acetonitrile: *V* water = 1:1). The dry extract was vortexed for 30 s and sonicated 10 min (4 °C water bath). After centrifuging for 15 min at 12000 rpm at 4 °C, the supernatant (60 μL) was transferred into a fresh 2 mL LC/MS glass vial for the UHPLC-QTOFMS analysis. Six biological replicates were performed for each tissue in this study.

### Analysis of non-volatile compounds in *P. cablin* tissues using UHPLC-QTOFMS

LC-MS/MS analyses were performed using an UHPLC system (1290, Agilent Technologies) with a UPLC BEH amide column (1.7 μm × 2.1 mm × 100 mm, Waters) coupled to TripleTOF 5600 (Q-TOF, AB Sciex). The mobile phase consisted of 25 mM NH_4_OAc and 25 mM NH_4_OH in water (pH=9.75) (A) and acetonitrile (B) was carried with elution gradient as follows: 0 min, 95% B; 7 min, 65% B; 9 min, 40% B; 9.1 min, 95% B; 12 min, 95% B, which was delivered at 0.5 mL min^-1^. The injection volume was 2 μL. The Triple TOF mass spectrometer was used for its ability to acquire MS/MS spectra on an information-dependent basis during an LC/MS experiment. Using this mode, the acquisition software (Analyst TF 1.7, AB Sciex) continuously evaluates the full scan survey MS data as it collects and triggers the acquisition of MS/MS spectra depending on preselected criteria. During each cycle, 12 precursor ions with an intensity greater than 100 were chosen for fragmentation at collision energy (CE) of 30 V (15 MS/MS events with each product ion accumulation for 50 msec). ESI source conditions were set as follows: Ion source gas 1 as 60 Psi, Ion source gas 2 as 60 Psi, Curtain gas as 35 Psi, source temperature 650 °C, Ion Spray Voltage Floating (ISVF) 5000 V or -4000 V in positive or negative modes, respectively.

### Data preprocessing and annotation

MS raw data files were converted to the mzXML format using ProteoWizard, and processed by R package XCMS (version 3.2). The preprocessing results generated a data matrix that included the retention time, mass-to-charge ratio (m/z) values, and peak intensity. The R package CAMERA was used for peak annotation after XCMS data processing. An in-house MS2 database was applied for metabolites identification. A data matrix, including the metabolite name (putatively identified by UHPLC-QTOFMS), sample information (six biological repeats for each sample), and raw abundance (peak area for each putatively identified metabolite), was generated, uploaded to MetaboAnalyst 3.0, and analyzed according to the instructions provided. On the Data Normalization page, we selected “Normalization by sum” for sample normalization, “None” for data transformation, and “Auto-scaling” for data scaling. The filtered data were loaded into Metabo-Analyst 3.0 for multivariate statistical analyses, including principal components analysis (PCA) and partial least squares discriminant analysis (PLS-DA). Discriminating compounds were then validated using a false discovery rate (FDR) test with P < 0.05, and the thresholds of fold-change ≥ 2 and q-value ≤ 0.05 for metabolites were considered to indicate differentially expressed metabolites. Differentially metabolites were analyzed by hierarchical clustering, and similarity assessment for clustering was based on the Euclidean distance coefficient. KEGG (http://www.kegg.jp/) was used to analyze the metabolic pathway.

## Results

### Determination of the volatile compounds in *P. cablin* leaves, stems, and flowers using GC-MS

**Figure 1:**
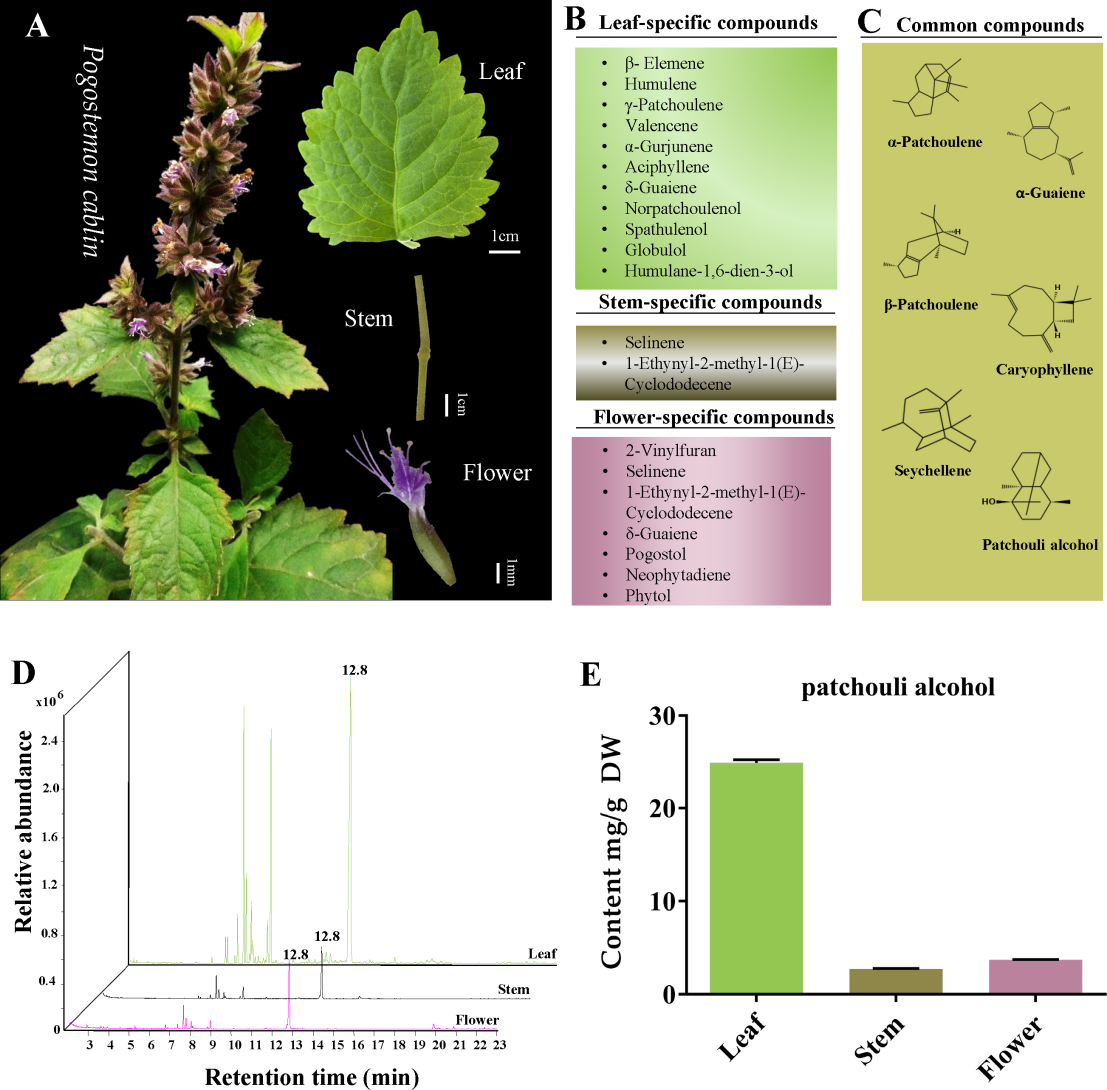
The tissue characteristics and volatile components analysis of *Pogostemon cablin*. **(A)** The phenotypic characteristics of *P. cablin* plants and their leaf, stem and flower. **(B)** Specific compounds in leaf, stem and flower. **(C)** Common compounds in leaf, stem and flower. **(D)** Gas chromatography-mass spectrometer (GC-MS) chromatograms of samples from the leaf, stem and flower, the retention time of patchouli alcohol is 12.8 min. **(E)** The content of patchouli alcohol in leaf, stem and flower. Error bars represent 95% confidence intervals. DW stands for dry weight.

It is well-acknowledged that *P. cablin* rarely blooms. Fortunately, in the present study, *P. cablin* blossomed in spring, and the characteristics of leaf, stem and flower tissues were recorded (**Figure 1A**). The leaf of *P. cablin* is broadly ovate, with irregular tooth fissure. The stem is covered with villus; the verticillaster has ten to more flowers, forming a spike, and the corolla is purple. Then the tissues were collected, and GC-MS was selected to detect and quantify the volatile compounds present in *P. cablin*. Differences were observed in the volatile compounds in the three tissues of *P. cablin*. 11, 2 and 7 specific volatile compounds (**Figure 1B**) and 6 common compounds (**Figure 1C**) were identified in leaf, stem and flower tissues, respectively. 6 common compounds were as follows: α-patchoulene, β-patchoulene, seychellene, α-guaiene, caryophyllene and patchouli alcohol. As shown in **Figure 1**, δ-Guaiene was detected in leaves and flowers, while selinene and 1-ethynyl-2-methyl-1(E)-cyclododecene were found in stems and flowers. Furthermore, 10 compounds, including β-elemene, humulene, γ-patchoulene, valencene, α-gurjunene, aciphyllene, norpatchoulenol, spathulenol, globulol and humulane-1,6-dien-3-ol, were specifically detected in leaves. Moreover, 4 compounds, including 2-vinylfuran, pogostol, neophytadiene and phytol, were exclusively detected in flower tissues. All compounds found in leaves and stems were sesquiterpenes or derivatives, while two diterpenes (neophytadiene and phytol) were identified in flowers.

The volatile compounds released from all three selected tissues suggested that patchouli alcohol is the primary compound in *P. cablin*. To accurately measure the content of patchouli alcohol in leaves, stems and flowers, the calibration curve generated with the patchouli alcohol standard was linear in the 0.01~1.14 mg/mL range, and the correlation coefficient was 0.99. GC-MS revealed the highest levels of patchouli alcohol in the leaves (24.89 ± 0.40 mg/g DW). On the contrary, the content of patchouli alcohol in stems (2.72 ± 0.07 mg/g DW) and flowers (3.71 ± 0.07 mg/g DW) was lower than in leaves (**Table S1**; **Figure 1D, E**).

### Metabolite assessment in leaves, stems, and flowers using UHPLC-QTOFMS

**Figure 2:**
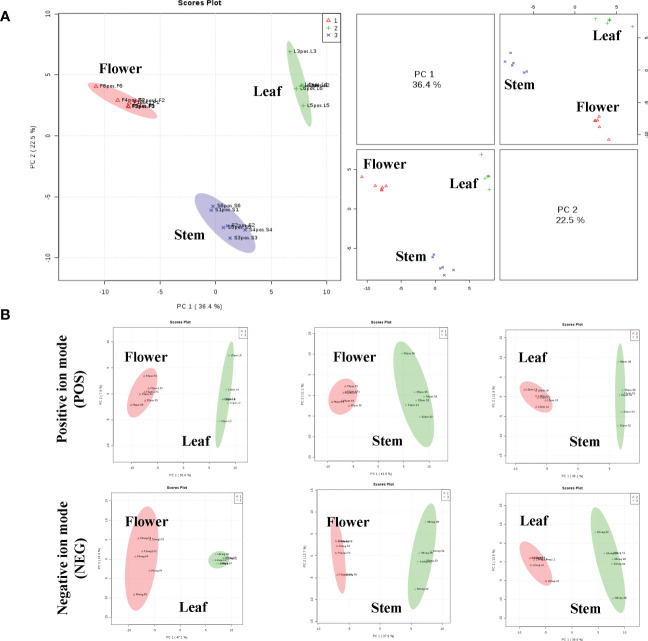
The principal component analysis (PCA) scatter plots between the three tissues of *P. cablin*. **(A)** PCA scatter plot of leaf (green circle), stem (blue circle) and flower (red circle) in positive ion mode (POS). **(B)** The PCA between any two tissues in *P. cablin* in positive and negative ion modes, respectively. The PCA was depends on the LC-MS data from all groups in positive and negative ion modes.

To analyze the non-volatile components and metabolism of *P. cablin*, UHPLC-QTOFMS was used to evaluate the abundance of metabolites in the leaves, stem and flower tissues of *P. cablin*. A total of 2817 and 3250 relative retention time-quantitative peaks were generated by positive and negative ion modes analyses in *P. cablin* (**Figure S1**). The raw abundance of each metabolite was calculated based on the peak area. Data were normalized using an internal standard (2-chloro-L-phenylalanine, Shanghai Hengbai Biotech Co., Ltd.), and the mean of the normalized peak area data was log2 transformed. After data normalization (**Figure S2**), principal component analysis (PCA) was performed. The PCA plot exhibited an obvious separation pattern among tissues in both ion modes (**Figure 2**). The first and second principal component (PC1 and PC2) scores of three different tissues were 36.4% and 22.5%, respectively (**Figure 2A**). As shown in **Figure 2B**, PC1 and PC2 accounted for 55.6% and 7.8% of the total variance within the flower-leaf group in positive ion mode (POS) and 47.1% and 10.6% in negative ion mode (NEG). Furthermore, PC1 and PC2 of the flower-stem group were 43.8% and 11.1% in POS mode and 37.9% and 12.7% in NEG mode, while the PC1 and PC2 of the leaf-stem group were 39.1% and 11.9% in the POS mode and 39.6% and 13.5% in the NEG mode. The results showed significant differences between groups, suggesting significant differences in metabolites among the flower-leaf, flower-stem, and leaf-stem groups.

### Metabolite variation assessed by PLS-DA, UVA, and clustering analysis

**Figure 3:**
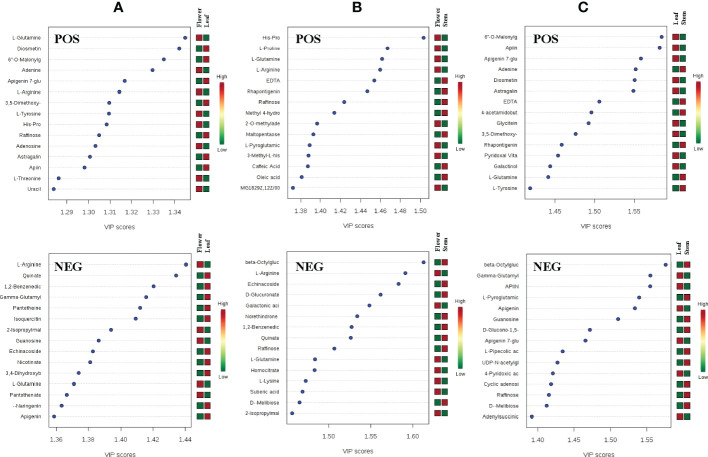
The VIP scores of compounds with high principal component ranking in the flower-leaf group **(A)**, flower-stem group **(B)** and leaf-stem group **(C)** of *P. cablin* in positive and negative ion modes. The color intensity represents the relative abundance of metabolites detected.

To further analyze the differences of metabolites in leaves, stems and flowers of *P. cablin*, partial least squares discriminant analysis (PLS-DA) was used to examine differences in secondary metabolism. Unlike PCA, PLS-DA is a supervised discriminant analysis method that uses partial least squares regression to simulate the relationship between metabolite expression levels. Features with variable importance in the projection (VIP) scores greater than 1 are important for the projection of the first principal component of the PLS-DA model and can be used as the criteria for feature screening. In our study, metabolites with a VIP >1 in the PLS-DA model were identified, and the top 15 most important metabolite compounds ranked by VIP scores are shown in **Figure 3**. By comparing the top 15 candidate compounds in the positive and negative ion modes between tissues, it was found that L-glutamine and raffinose had high VIP scores in leaf, stem and flower, which could be used to distinguish the three tissues. Compounds more important in leaf than stem and flower included gamma-glutamylcysteine, guanosine, apigenin, diosmetin, 6”-o-malonylglycitin, adenine, apigenin 7-glucoside, 3,5-dimethoxy-4-hydroxycinnamic acid, L-tyrosine, astragalin, and apiin. Compounds more important in flower than leaf and stem included L-arginine, quinate, 1,2-benzenedicarboxylic acid, 2-isopropylmalic acid, echinacoside, and His-Pro. Moreover, compounds more important in the stem than in the leaves and flowers included beta-octylglucoside, D-melibiose, EDTA, rhapontigenin, and L-pyroglutamic acid (**Figure 3**).

Univariate analysis (UVA) can objectively reflect the significance of metabolite changes between the two samples. In this study, a P-value less than 0.05 and | Log2 (FC) |>1 were used for screening differential compounds. The results were illustrated in volcano plots (**Figure S3**). 65 and 61 differential compounds were identified between flower and leaf tissues using the POS and NEG modes (**Figure S3A**); 48 and 44 differential compounds were identified between flower and stem tissues using the POS and NEG modes (**Figure S3B**), while 46 and 49 differential compounds were identified between leaf and stem tissues using the POS and NEG modes, respectively (**Figure S3C**). Subsequently, the differentially abundant metabolites obtained in any two tissues by POS and NEG modes were analyzed by hierarchical clustering (**Figure S4**). Each shade represents the relative expression level of a certain metabolite in each tissue sample. Overall, significant metabolite differences were observed between leaf, stem and flower groups. By combining the results of the POS and NEG modes, 112, 77 and 83 different metabolites between the flower-leaf (**Table S2**), flower-stem(**Table S3**), and leaf-stem (**Table S4**) groups were identified respectively, indicating that the difference in metabolite abundance between flower and leaf tissues was significantly. Among all metabolites examined, 33 compounds from leaves were found to be different in both the flower and stem groups, while no significant differences were found between flowers and stems. The 33 compounds differentially expressed in leaves can be used as potential metabolite biomarkers of *P. cablin* leaves. Similarly, 32 and 12 potential specific metabolite biomarkers for flowers and stems were found in this study. In addition, 22 common compounds were found in leaf, stem and flower tissues, including raffinose and adenine, which exhibited highly concentrations in the three tissues (**Table 1** and **Figure S5**).

**Table 1:** The specific and common metabolite compounds of different tissues detected by UHPLC-QTOFMS in *P. cablin*.

NO.	Flower DEM compounds (32) ^a^	Leaf DEM compounds (33) ^b^	Stem DEM compounds (12) ^c^	Common compounds (22) ^d^
1	L-Arginine	Diosmetin	EDTA	L-Glutamine
2	L-Proline	6’’-O-Malonylglycitin	Rhapontigenin	Adenine
3	Glyceryl Monolinoleate	Apigenin 7-glucoside	S-Adenosylmethionine	His-Pro
4	2-Amino-2-methyl-1,3-propanediol	3,5-Dimethoxy-4-hydroxycinnamic acid	Histamine	Raffinose
5	1-Methylhistidine	L-Tyrosine	DL-3-Hydroxybutyric acid	Apiin
6	3-Methyl-L-histidine	Adenosine	3-Methylindole	L-Threonine
7	3-Methylhistidine	Astragalin	D-Mannose	Uracil
8	Nicotinate	Glycitein	Xanthosine	Pantothenate
9	Arachidonic Acid peroxide free	6-Hydroxydopamine	L-Pipecolic acid	L-Pyroglutamic acid
10	Cytidine 2’,3’-cyclic phosphate	4-Acetamidobutanoate	Galactonic acid	Caffeic Acid
11	1,2-Benzenedicarboxylic acid	Glycerophosphocholine	alpha-Linolenic acid	Maltopentaose
12	L-NG-Monomethylarginine	Riboflavin	D-galacturonic acid	Larixinic Acid
13	DL-Indole-3-lactic acid	Malvidin 3-O-glucoside cation		L-Tryptophan
14	Quercetin 3’-methyl ether	Phosphorylcholine		Allantoate/Allantoic acid
15	Methyl 4-hydroxybenzoate	Galactinol		D–Melibiose
16	S-Methyl-5’-thioadenosine	Lys-Asp		Echinacoside
17	1-Oleoyl-sn-glycero-3-phosphocholine	Stearidonic Acid		3,4-Dihydroxybenzoate Protocatechuic acid
18	Quinate	N-.alpha.-Acetyl-L-arginine		Apigenin
19	Pantetheine	1,2-dihexadecanoyl-sn-glycero-3-phosphocholine		L-Cysteic acid
20	Isoquercitin	Thioetheramide-PC		Adenylsuccinic acid
21	2-Isopropylmalic acid	Abscisic Acid cis,trans		Oleanolic acid
22	1-Palmitoyl-2-linoleoyl-sn-glycero-3-phosphate	Gamma-Glutamylcysteine		beta-Octylglucoside
23	L-Lysine	4-Pyridoxic acid		
24	Homocitrate	UDP-N-acetylglucosamine		
25	cis-6,9,12-Linolenic acid	Adipic acid		
26	Suberic acid	D-Glucono-1,5-lactone		
27	D-Glucuronate	Indapamide		
28	Coumestrol	Acetyl-DL-Valine		
29	cis-Aconitate	Maslinic Acid		
30	4-Acetoxyphenol	ketoisocaproic acid		
31	L-Asparagine	Folinic acid		
32	Cytidine 5’-monophosphate CMP	Phenylpyruvate		
33		11-Keto-.beta.-boswellic acid		

^a^significantly differentially expressed in flower compared with leaf and stem, ^b^Significantly differentially expressed in leaf compared to flower and leaf and ^c^significantly differentially expressed in stem compared with flower and leaf, ^d^Common expressed in three types of tissue samples. DEM stands for differential expression of metabolites.

### Pathway identification of differential metabolites

**Figure 4:**
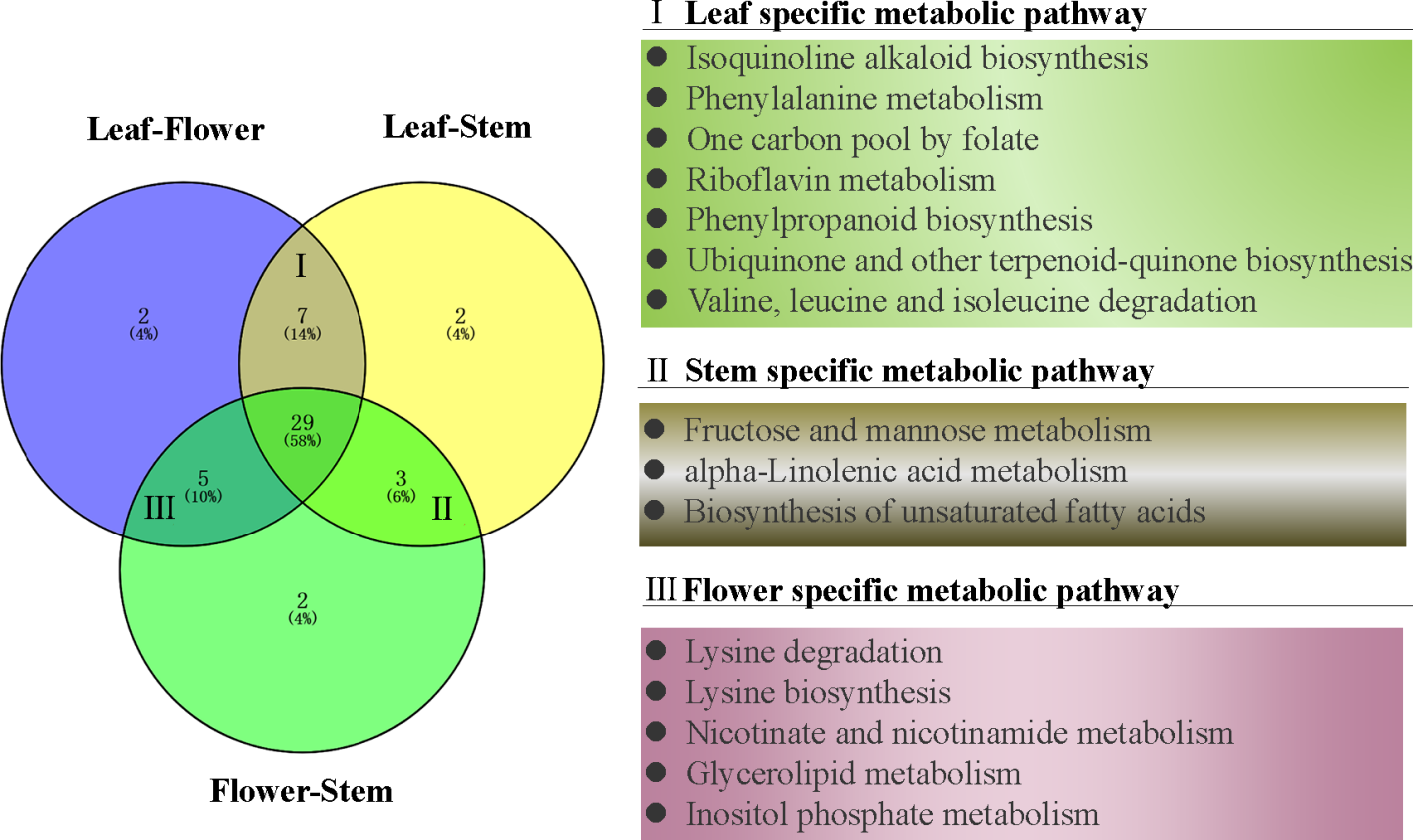
The venn diagram of differential metabolic pathways in *P. cablin.* The pathways were referenced in kyoto encyclopedia of genes and genomes (KEGG).

To further understand the metabolic pathways and specific biological functions of leaf, stem and flower metabolites in *P. cablin*, Pubchem and the Kyoto encyclopedia of genes and genomes (KEGG) databases were used in this study. Pathway significant enrichment analysis takes KEGG pathway as a unit, and applies hypergeometric test to find out the pathway that is significantly enriched in the differential metabolite compared with the background of the whole metabolome. KEGG, PubChem and other authoritative metabolite databases were used to analyze the differential metabolites and KEGG analysis was conducted using the screening criteria P-value less than 0.05 and | Log2 (FC) |>1. After obtaining the matching information of different metabolites, we searched the pathway database and analyzed the metabolic pathways. 50 metabolic pathways were significantly dysregulated in paired tissue comparisons (**Figure 4**), and 29 exhibited differences between leaf, stem and flower tissues (**Table S5**). 7 metabolic pathways were differentially expressed in leaf tissues, including isoquinoline alkaloid biosynthesis, phenylalanine metabolism, one carbon pool by folate, riboflavin metabolism, phenylpropanoid biosynthesis, ubiquinone and other terpenoid-quinone biosynthesis and valine, leucine and isoleucine degradation. Similarly, 3 individual metabolic pathways in the stem, including fructose and mannose metabolism, alpha-linolenic acid metabolism, and biosynthesis of unsaturated fatty acids, were differentially expressed compared with flower or leaf tissues. 5 metabolic pathways were differentially expressed in the flower tissues compared with stem or leaf tissues, including lysine degradation, lysine biosynthesis, nicotinate and nicotinamide metabolism, glycerolipid metabolism and inositol phosphate metabolism (**Figure 4**).

### Integration of pathways and differential metabolites from various tissues

**Figure 5:**
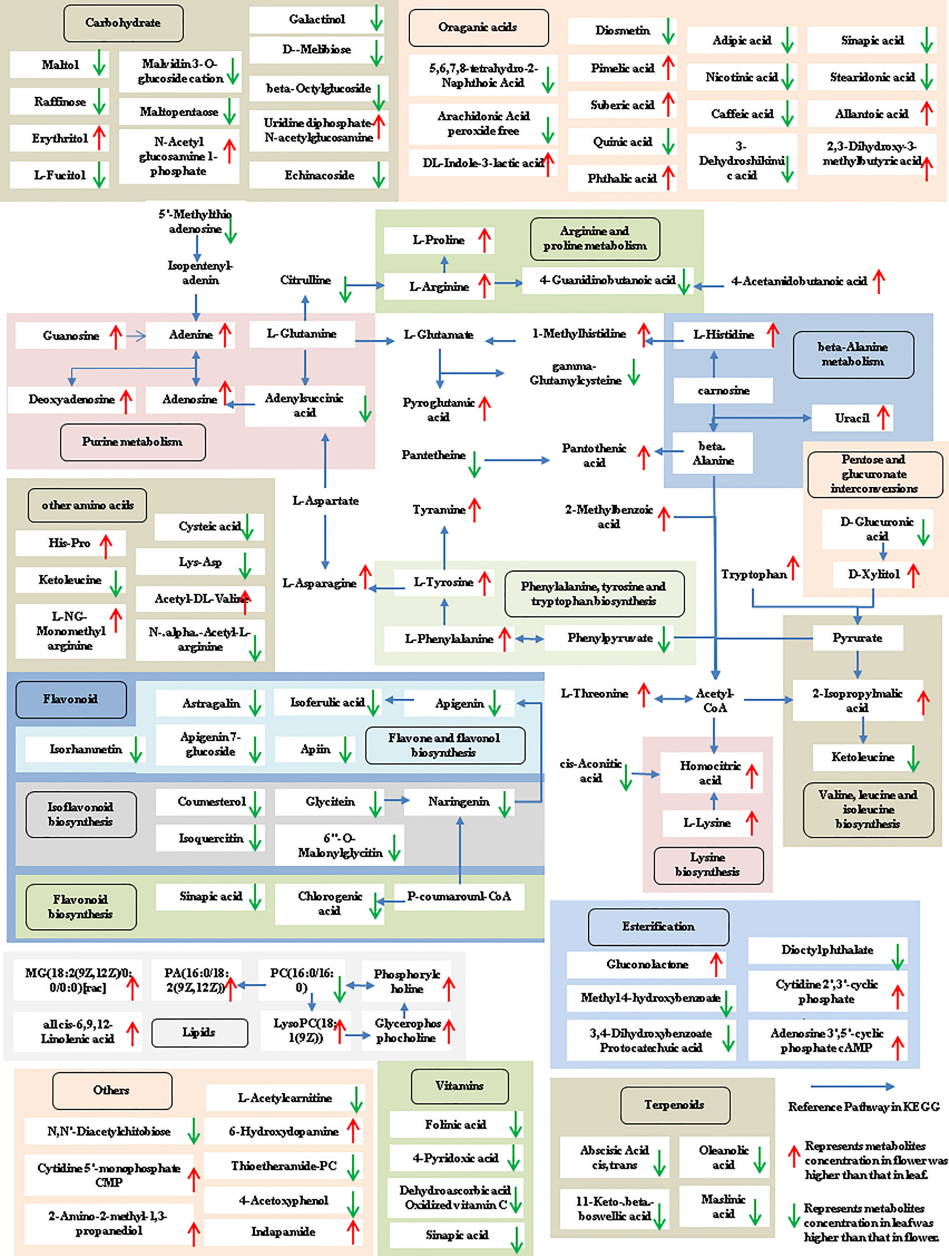
Metabolic pathways analysis in flower-leaf group of *P. cablin*. The 112 metabolites identified in flower-leaf group were mapped onto primary and secondary metabolism. The upward-pointing red arrows represent the value of |log2| (content flower/leaf) >1, which means the level of metabolites in flower is higher than that in leaf, the value of |log2|(content flower/leaf) <1 means the level of metabolites in leaf was higher than that in flower, and represented by downward-pointing green arrows.

**Figure 6:**
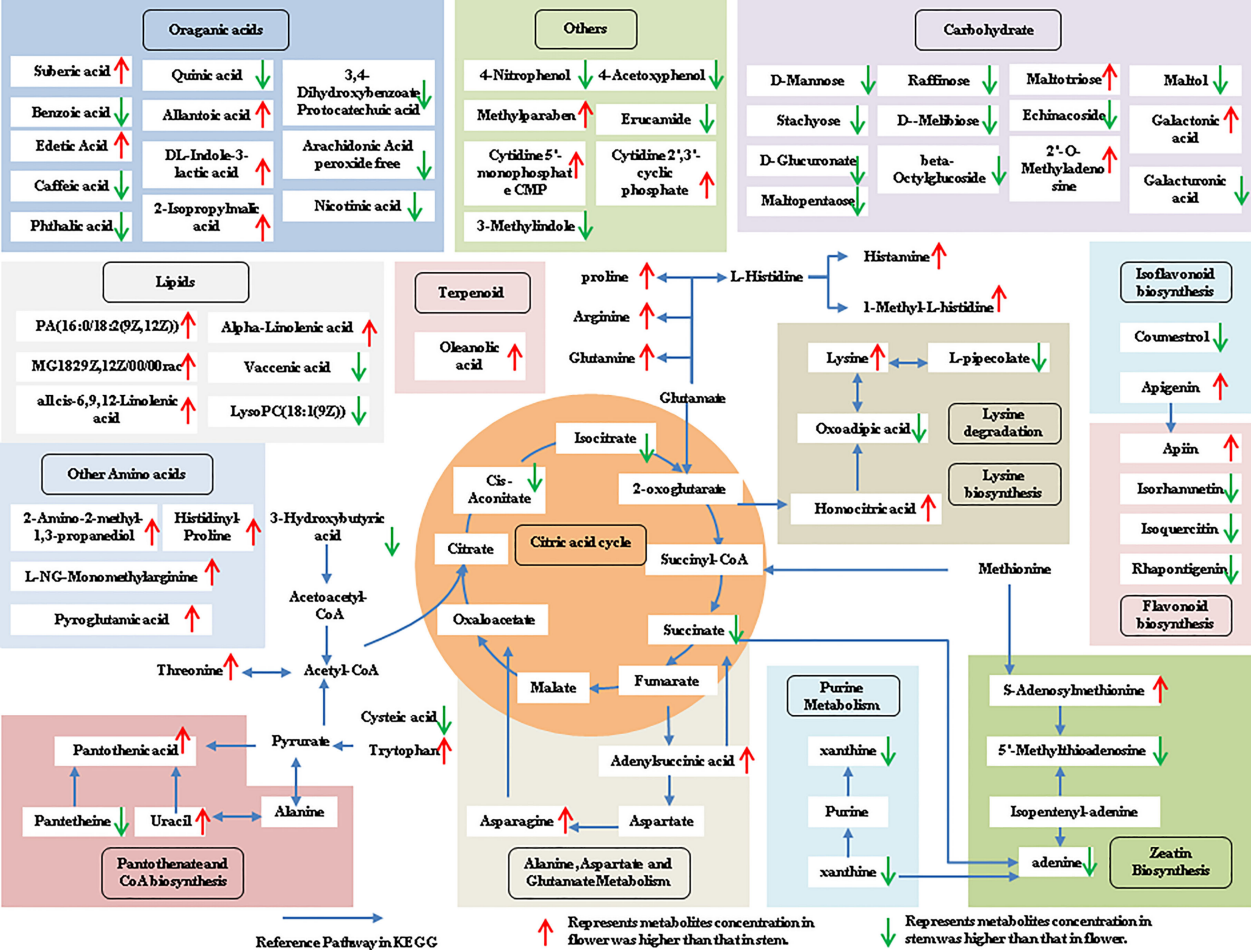
Metabolic pathways analysis in flower-stem group of *P. cablin*. The 77 metabolites identified in flower-stem group were mapped onto primary and secondary metabolism. The upward-pointing red arrows represent the value of |log2| (content flower/stem) >1, which means the level of metabolites in flower is higher than that in stem, the value of |log2|(content flower/stem) <1 represents metabolites concentration in stem was higher than that in flower, and represented by downward-pointing green arrows.

**Figure 7:**
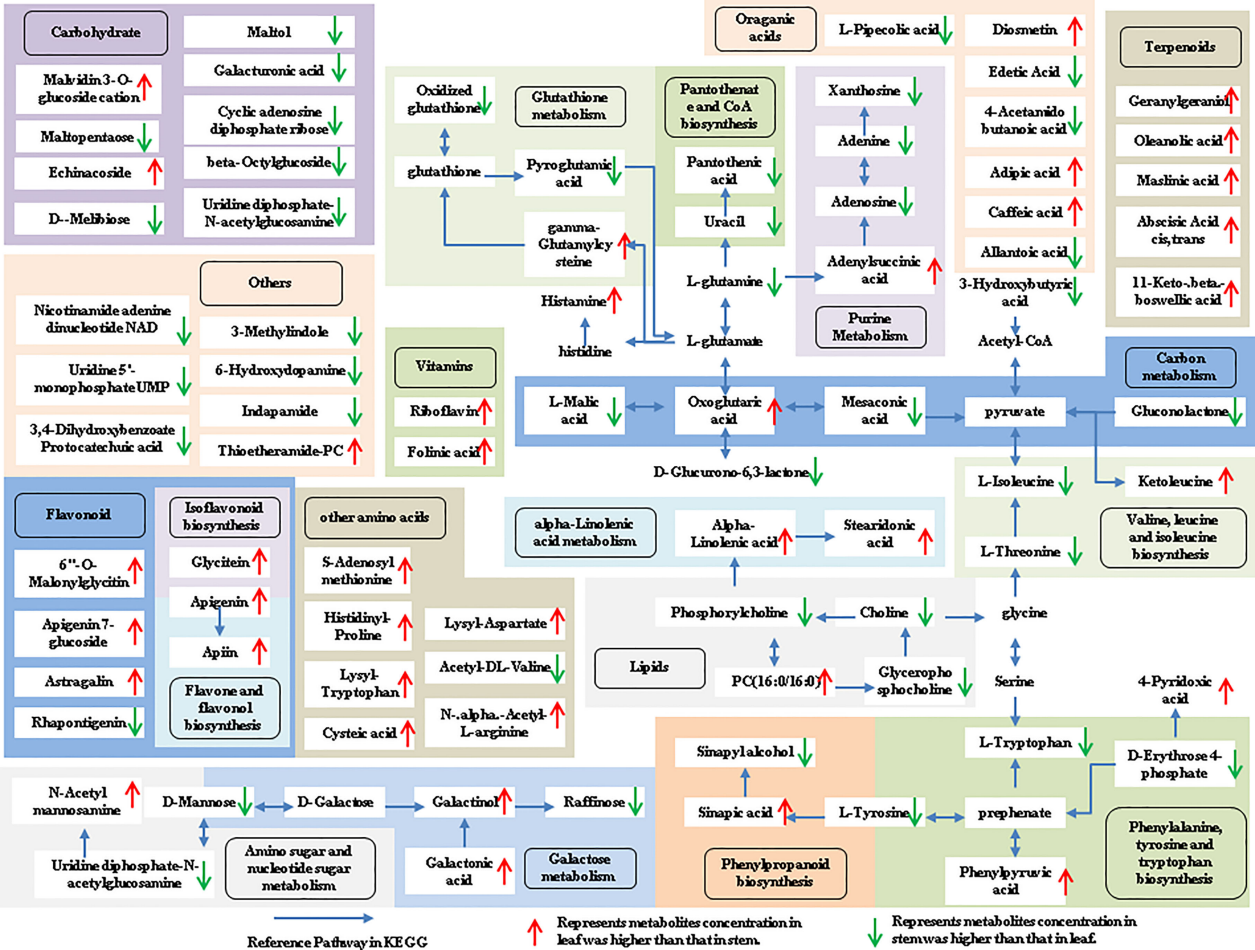
Metabolic pathways analysis in leaf-stem group of *P. cablin*. The 83 metabolites identified in leaf-stem group were mapped onto primary and secondary metabolism. The upward-pointing red arrows represent the value of |log2| (content leaf/stem) >1, which means the level of metabolites in leaf is higher than that in stem, the value of |log2|(content leaf/stem) <1 represents metabolites concentration in stem was higher than that in leaf, and represented by downward-pointing green arrows.

The tissue-specific expression patterns of differentially expressed metabolites identified in the flower-leaf, flower-stem and stem-leaf groups were integrated and mapped onto the most relevant metabolic pathways, including both primary and secondary metabolic pathways (**Figures 5**-**7**). A comparison of leaf and flower tissues revealed that 112 metabolites were enriched in 43 pathways. As illustrated in **Figure 5**, the flower contained higher levels of amino acids (e.g., L-proline and L-lysine), lipids (e.g., PA, as well as cis-6, 9, 12-linolenic acid) and purines (e.g., adenine). In contrast, leaves contained higher levels of terpenoids (e.g., oleanolic acid), vitamins (e.g., folinic and sinapic acid), flavonoids (e.g., apigenin and naringenin), most carbohydrates (e.g., raffinose and D-melibiose) and organic acids (e.g., diosmetin). 77 differentially expressed metabolites were identified between stem and flower, and these metabolites were enriched in 39 metabolic pathways. As shown in **Figure 6**, the flower contained higher levels of amino acids (e.g., L-proline and L-lysine) and lipids (e.g., PA as well as cis-6, 9, 12-linolenic acid), stems contained higher levels of purines (e.g., adenine), flavonoids (e.g., apigenin and naringenin), and most carbohydrates (e.g., raffinose and D-melibiose). In addition, 83 differentially expressed metabolites between stem and leaf were enriched in 41 pathways. As shown in **Figure 7**, the leaf contained higher levels of terpenoids (e.g., oleanolic acid and maslinic acid), dipeptide and peptide amino acids (e.g., lysyl-aspartate and cysteic acid), vitamins (e.g., folinic acid and riboflavin) and flavonoids (e.g., apigenin and astragalin), the stem contained higher levels of purines (e.g., adenine), amino acids (e.g., L-isoleucine and L-glutamine), lipids (e.g., adenine) and carbohydrates (e.g., maltol and D-melibiose).

**Figure 8:**
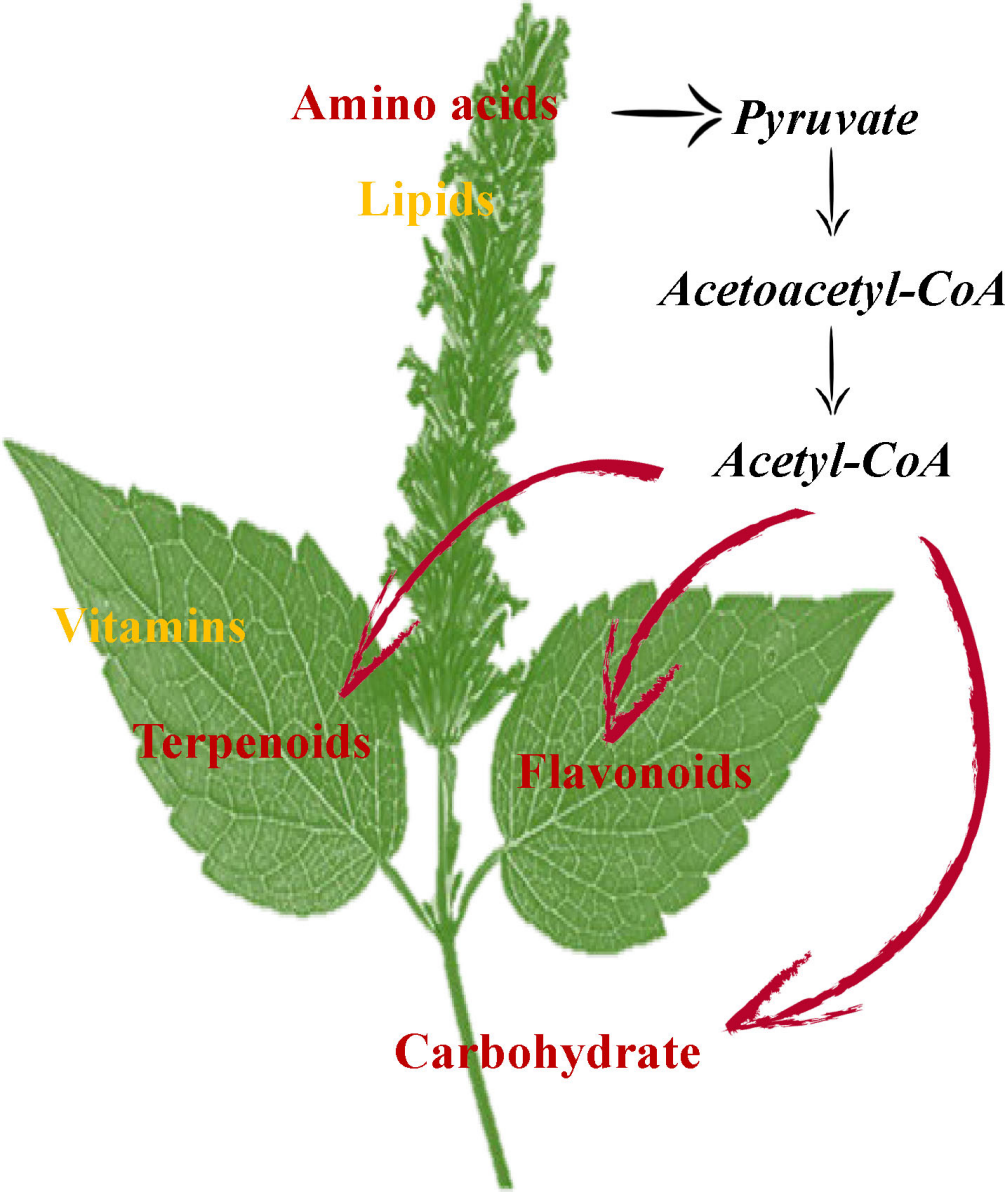
The possible mechanisms for the synthesis and accumulation of compounds in the leaves, stems and flowers of *P. cablin*. Flowers contain high levels of amino acids and lipids. Leaves contain high levels of terpenoids and flavonoids. Stems contain high levels of carbohydrates.

In conclusion, GC-MS and UHPLC-QTOFMS analysis revealed the accumulation and enrichment pathways of different metabolites among leaves, stems and flowers. Our findings suggest that the flowers accumulate more lipids and amino acids, including proline, lysine, arginine, asparagine, threonine, and tryptophan in *P. cablin*. Meanwhile, higher levels of terpenoids, vitamins and flavonoids accumulated in leaves, while higher levels of carbohydrates were found in stems. The oxidative decarboxylation of pyruvate and the catabolism of amino acids (such as lysine and tryptophan) produce acetyl-CoA, which is also a precursor involved in the biosynthesis of terpenoids, flavonoids and carbohydrates. The above results provide compelling evidence of the exchange of metabolites among leaf, stem and flower tissues in *P. cablin* and indicate a potential complex metabolic network in *P. cablin* (**Figure 8**).

## Discussion

*P. cablin* is widely cultivated in many countries, including China, India, Malaysia, Indonesia, etc. It is rich in patchouli oil and represents the only natural source of essential oil. Moreover, *P. cablin* is one of the 20 essential oil plants most commonly traded in the international market, with huge market demand. Given the absence of a synthetic substitute, patchouli oil exhibits an increasingly important market value (Swamy et al., 2015). In addition to volatile substances such as essential oil, *P. cablin* is also rich in non-volatile compounds, such as flavonoids (Li et al., 2011). In recent years, most emphasis has been placed on the pharmacological activity and biosynthetic mechanism of metabolites in *P. cablin*, and there is no in-depth discussion on the distribution and exchange of compounds in key tissues of *P. cablin*, such as leaves, stems and flowers. Our research systematically analyzed the distribution and types of volatile and non-volatile substances in the leaves, stems and flowers of *P. cablin* and the metabolic pathways involved in them through GC-MS and UHPLC-QTOFMS. At the same time, the exchange of metabolites between different tissues of *P. cablin* was analyzed, providing a reference for future studies on the metabolic network of *P. cablin*.

Although the metabolites in the leaves and stems of *P. cablin* have been extensively reported, metabolites in the flowers of *P. cablin* have been understudied since they rarely bloom. Fortunately, in the present study, enough flowers were collected in spring. To conduct an integrated analysis of the differences in metabolite abundance in the three tissues, GC-MS was selected to analyze the volatile compounds. The results revealed that the leaf, stem and flower tissues of *P. cablin* were rich in various volatile components, and the leaves contained more volatile substances than the stems and flowers. It was found that patchouli alcohol is the main compound in the three tissues. The quantitative results showed that patchouli alcohol exhibited the highest levels in leaves (24.89 mg/g), 9.12 and 6.69-fold higher than in stems and flowers. It has been reported that glandular hair is the main site of volatile oil accumulation, and a large amount of patchouli alcohol in patchouli leaves is related to its rich epidermal hair (Guo et al., 2013), which warrants further study. In addition, as the representative of sesquiterpenes in *P. cablin*, patchouli alcohol is mainly generated through the MVA and MEP pathways, and the original substrate of the MVA pathway is acetyl coenzyme A (Li et al., 2021). We have reason to believe that acetyl coenzyme A exists in three tissues and participates in the biosynthesis of volatile substances of patchouli, such as terpenoids.

In addition to volatile compounds, a variety of non-volatile compounds is present in *P. cablin*. It has been reported that the dried aerial parts of *P. cablin* contain 33 flavonoids, 21 organic acids and 9 phenylpropanoids, ect (Xie et al., 2022), but they are limited to the analysis of mixed tissues. UHPLC-QTOFMS can comprehensively predict the main active components in the leaves, stems and flowers of *P. cablin* under positive and negative ion scanning modes. PCA analysis between the flower-leaf, flower-stem and leaf-stem groups of *P. cablin* was conducted to obtain the scores of PC1 and PC2 in the positive and negative ion mode, respectively. Each tissue can be divided into different regions independently. It was found that the differences between groups were significant in the top-ranked components, indicating that there are significant differences in the main metabolites between the flower-leaf, flower-stem and leaf-stem. The Univariate analysis further identified the different metabolites among groups, including 33 compounds in leaves and 32 in flowers. However, there were fewer individual compounds in stems (n=12). These compound molecules can be used as biomarkers for three tissues of *P. cablin* for germplasm identification and quality evaluation, which may be attributed to the fact that *P. cablin* is dependent on asexual reproduction, resulting in a single genetic background.

KEGG analysis revealed flower-specific pathways, including lysine degradation, lysine biosynthesis, nicotinate and nicotinamide metabolism, glycerolipid metabolism and inositol phosphate metabolism. It was found that flowers contained high levels of lipids and amino acids, such as proline and lysine. However, there were no differences in proline abundance between leaves and stems. A study reported that the proline level in tomato flowers was 60 times higher than that in other tissues (Schwacke et al., 1999), consistent with our findings, indicating that the accumulation of proline is related to the growth and development of the reproductive organs of *P. cablin*. Indeed, as the reproductive organs of plants, flowers contain more lipids than other organs (Wang et al., 2016). Higher lipid levels in *P. cablin* indicate a certain resistance to environmental stress. In addition, pogostol, neophytadiene, and phytol were detected only in flower tissues and may be associated with metabolic pathways specific to flowers. Neophytadiene and phytol are diterpenoids, and little research has been done on their biosynthesis. In our previous study, OEL4 and OEL12 transgenic tobacco plants, which overexpressed *PcFPPS*, exhibited significantly higher levels of phytol and neophytadiene than wild-type lines (Wang et al., 2022). Based on the synthetic pathways involved in terpenoid biosynthesis, diterpenoid biosynthesis is widely thought to be related to MEP synthesis. Studies have found that L-lysine is involved in carotenoid biosynthesis (Henke et al., 2018), however, carotenoids, chlorophyll, phytol, tocopherol and prenylated hydrazine share a common precursor produced by the plastid MEP pathway. We hypothesize that lysine can form precursors of the MEP pathway to synthesize some tetraterpenoids and sterols,such as carotenoids and phytol.

The results of GC-MS revealed that leaves contain more sesquiterpenoids compared with flowers or stems, β-elemene, humulene, (+)spathulenol, valencene, alpha-Gurjunene and aciphyllene were found exclusively in leaves. It is well known that sesquiterpenes are mainly synthesized from acetyl coenzyme A *via* the MVA pathway (Wang et al., 2017), and acetyl coenzyme A is crucial for many metabolic and decomposition pathways, such as fatty acid biosynthesis(Tang et al., 2018). Moreover, Acetyl coenzyme A is a key precursor of the triterpene squalene. The accumulation of acetyl coenzyme A can be promoted by increasing the synthesis of pyruvate, which can effectively increase the content of squalene (Huang et al., 2018). Overwhelming evidence substantiates that the biosynthesis of acetyl-CoA derivatives can be enhanced by providing sufficient precursors (Meadows et al., 2016). Therefore, acetyl coenzyme A may be directly used to form terpenoids, thereby reducing the formation of ketone bodies, leading to the accumulation of terpenoids and the reduction of lipid content.

KEGG analysis revealed that metabolite content related to the citrate cycle, glyox-ylate and dicarboxylate metabolism, and galactose metabolism significantly differed among tissue types. As the main pathway of sugar aerobic oxidation, the tricarboxylic acid cycle of plants is a main energy source for plants. The key regulatory enzyme of the TCA cycle is citrate synthase (CS), which regulates energy generation during mitochondrial respiration by catalyzing the reaction between oxaloacetic acid (OAA) and acetyl coenzyme A (Ac-CoA) to generate citrate and CoA (Li et al., 2016). It is well-established that stem tissues maintain high levels of carbohydrates through various synthetic pathways. Stems are formed by tissues responsible for transportation, storage, and support, and carbohydrates play important roles in each of these functions. Cellulose is a key component of the cell wall, which supports the elongation of the stem and is essential for plant anisotropic growth. (McFarlane et al., 2014). Moreover, maintaining high levels of carbohydrates in the stem represents another strategy to resist environmental stress. In a study on plant responses to desiccation, stem tissue accumulated sucrose and starch, unlike leaf tissue. Leaves died and were abscised to prevent water loss due to transpiration; however, stem photosynthesis was still observed, and stem tissues entered a dormant state until the period of water restriction ended (Liu et al., 2007). Overall, the present study’s findings emphasize the differential metabolites in the leaves, stems and flowers of *P. cablin* and the metabolic pathways involved, providing novel insights to explain the differences in metabolic components caused by tissue differences. Our findings are expected to promote research on the metabolic network of *P. cablin*, the diversified application of *P. cablin* and the innovation, sustainable development and utilization of *P. cablin* germplasm resources.

## Data availability statement

The original contributions presented in the study are included in the article/**Supplementary Material**. Further inquiries can be directed to the corresponding author.

## Author contributions

LC, LZ, and RZ conceived and designed the experiments. LZ, DW, JZ, DZ, HZ, and XBW performed the experiments. LZ, XZ, LG, XMW, LC, DW, and HZ analyzed the data. LC, LZ, and XBW wrote the manuscript. All authors contributed to the article and approved the submitted version.
