# Supplementary material for: GC-MS and UHPLC-QTOFMS-assisted identification of the differential metabolites and metabolic pathways in key tissues of Pogostemon cablin
Source: Front Plant Sci. 2023 Feb 27;14:1098280. doi: 10.3389/fpls.2023.1098280 (PMC10009150; doi:10.3389/fpls.2023.1098280)
Supplement: Supplementary file 1 [file DataSheet_1.pdf]

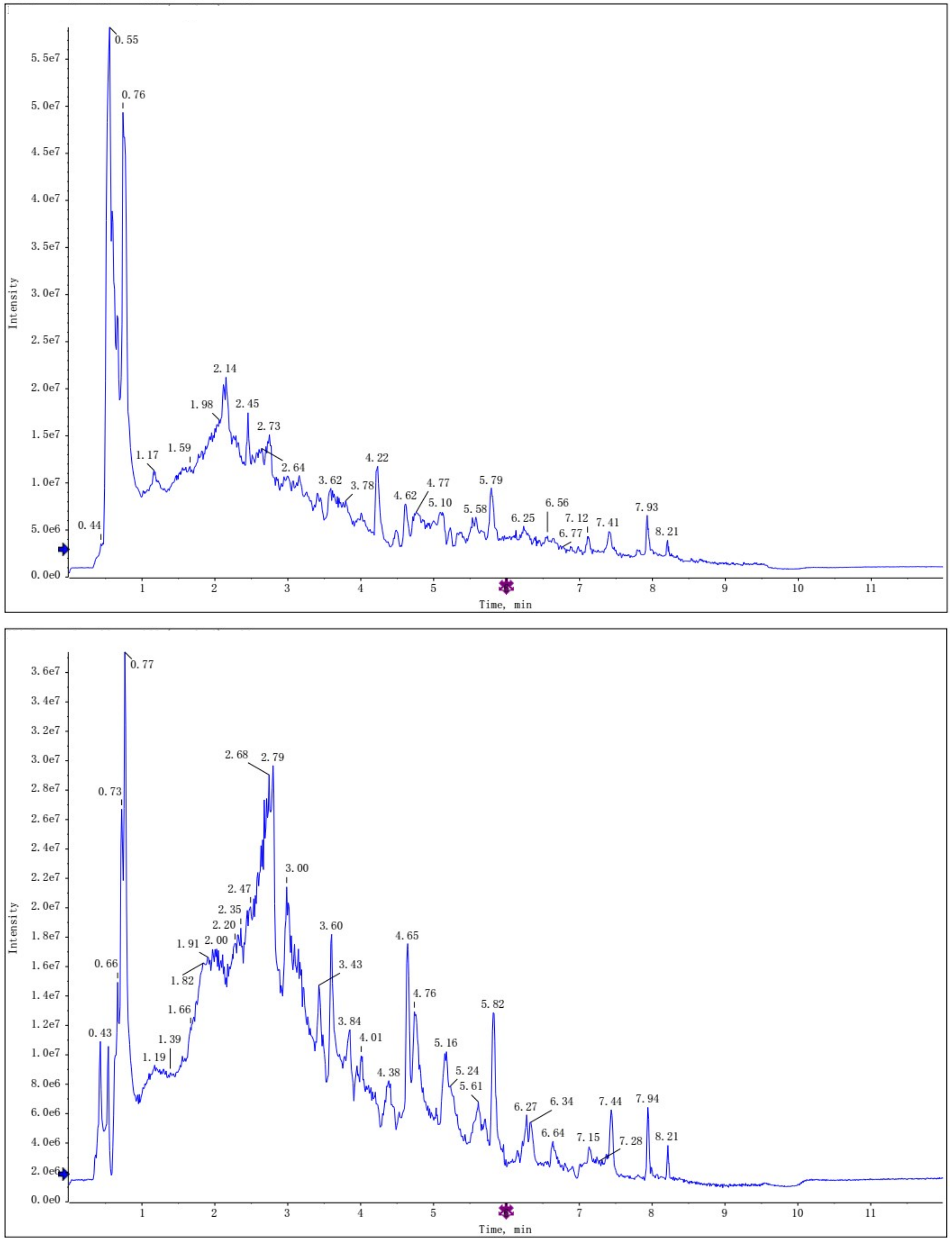


Figure S1. LC-MS chromatograms of QC samples in both positive and negative ion modes.

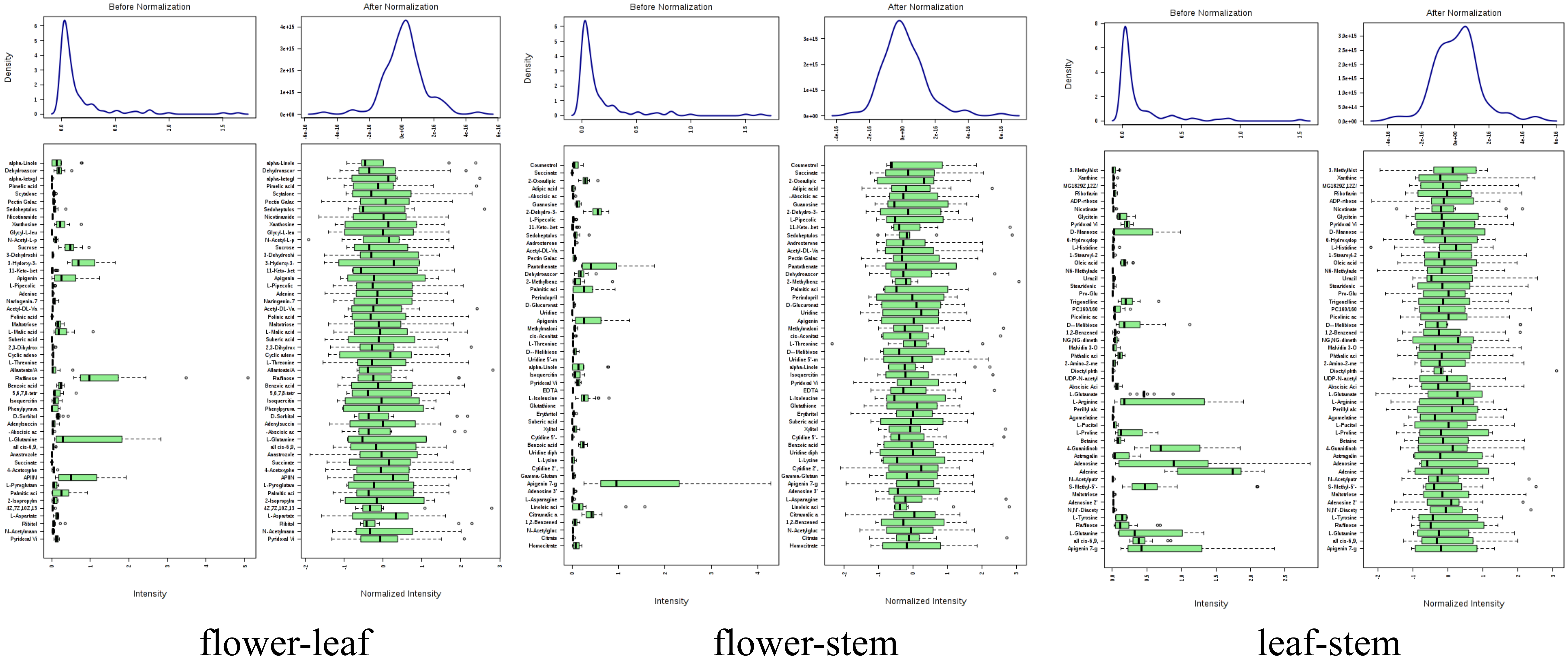


Figure S2. The data normalization result showing a graphical summary of the metabolites between flower-leaf, flower-stem and leaf-stem in *P. cablin* before and after the normalization procedures.

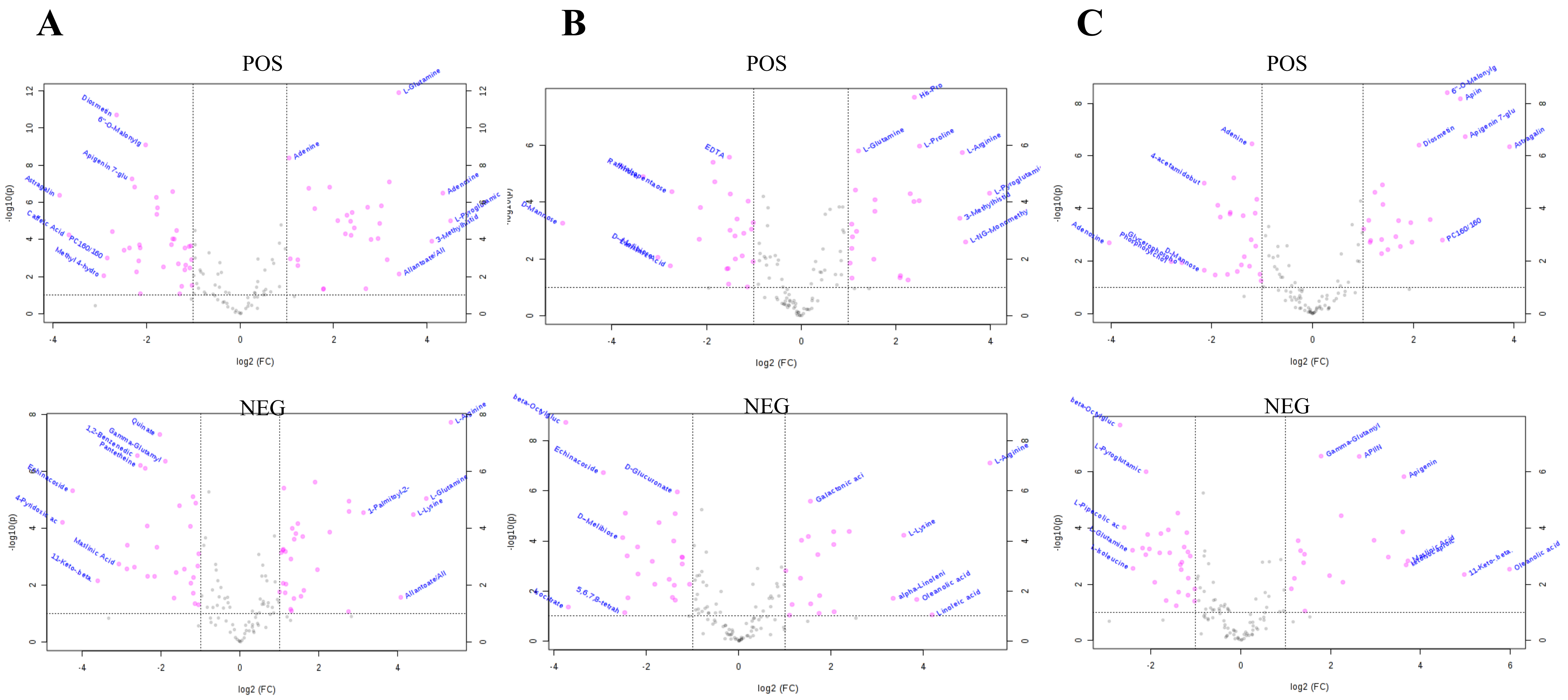


Figure S3. Volcano plot of flower-leaf group(A), flower-stem group(B) and leaf-stem group(C) of *P. cablin* metabolome in positive and negative ion modes, respectively. The volcano plot analysis, features with a fold change >2 ( | log2 | FC >1) and P-value (t-test)<0.05 were included in the volcano plots. Red points above the dotted line represent different metabolites with of any two tissues of *P. cablin* in positive and negative ion modes, respectively.

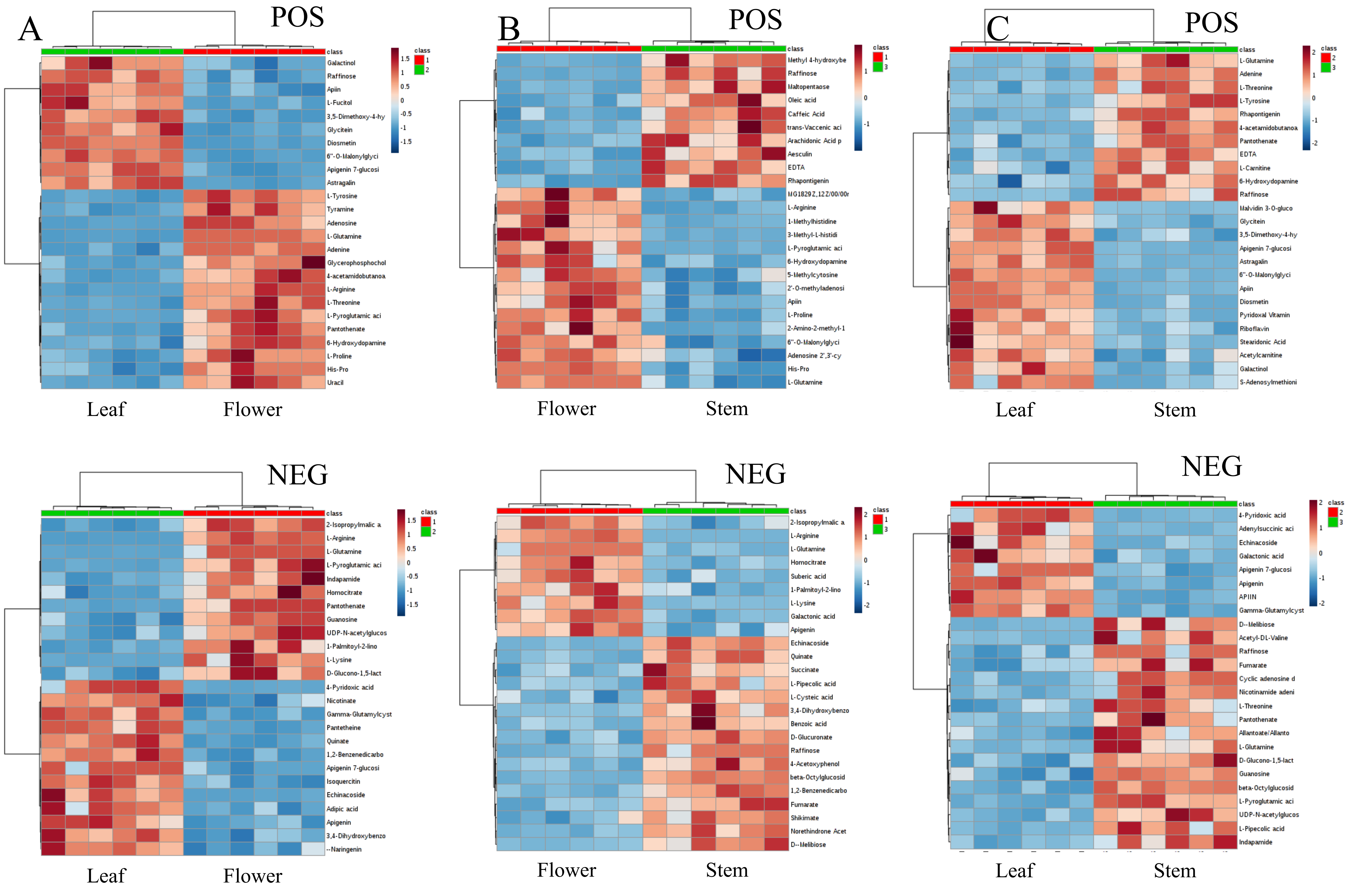


Figure S4. Cluster analysis of flower-leaf group(A), flower-stem group (B) and leaf-stem group(C) (T-test / ANOVA top 25) of *P. cablin* metabolome in positive and negative ion modes, respectively. Columns and rows represent individual metabolites and different samples, respectively. The colour intensity represents the relative abundance of metabolites detected.

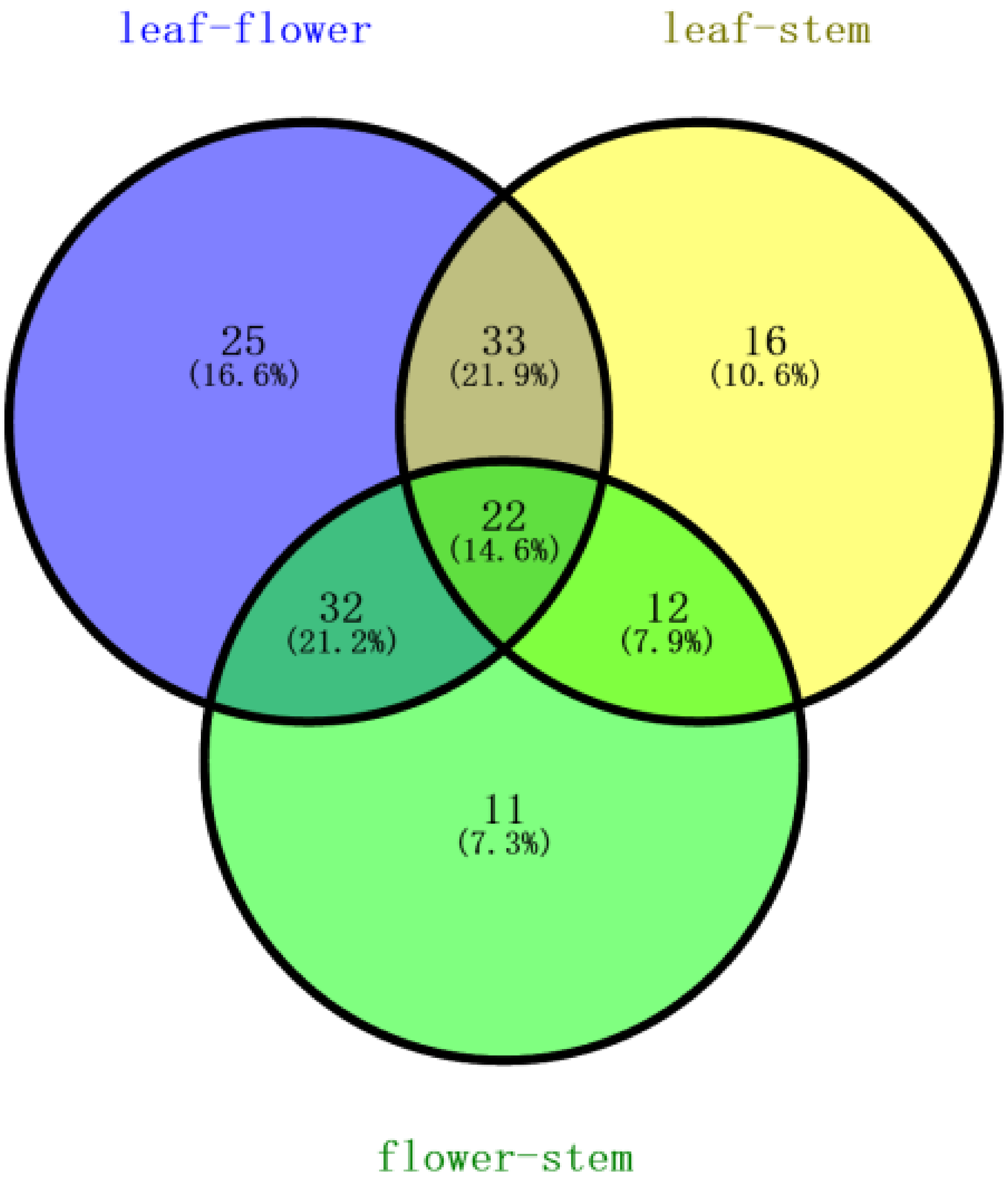


Figure S5. Venn diagram of differential metabolites detected in different tissues of *P. cablin* by UHPLC-QTOF-MS.
